# Kakkonto Inhibits Cytokine Production Induced by Rhinovirus Infection in Primary Cultures of Human Nasal Epithelial Cells

**DOI:** 10.3389/fphar.2021.687818

**Published:** 2021-08-31

**Authors:** Natsumi Saito, Akiko Kikuchi, Mutsuo Yamaya, Xue Deng, Mitsuru Sugawara, Shin Takayama, Ryoichi Nagatomi, Tadashi Ishii

**Affiliations:** ^1^ Department of Education and Support for Regional Medicine, Tohoku University Hospital, Sendai, Japan; ^2^ Department of Kampo and Integrative Medicine, Tohoku University Graduate School of Medicine, Sendai, Japan; ^3^ Department of Respiratory Medicine, Tohoku University Graduate School of Medicine, Sendai, Japan; ^4^ Department of Otolaryngology, Tohoku Kosai Hospital, Sendai, Japan; ^5^ Laboratory of Health and Sports Science, Division of Biomedical Engineering for Health and Welfare, Tohoku University Graduate School of Biomedical Engineering, Sendai, Japan

**Keywords:** kakkonto, Kampo, traditional herbal medicine, Rhinovirus, human airway epithelial cells, cytokine

## Abstract

Rhinovirus (RV) is a primary etiologic agent of common cold that can subsequently acutely exacerbate bronchial asthma or chronic obstructive pulmonary disease. Kakkonto (Ge-gen-tang in Chinese), one of the most frequently prescribed traditional Japanese (Kampo) medicines, is used for treating common cold, shoulder stiffness, or inflammatory diseases of the upper body. Previous experimental studies have indicated that kakkonto exerts antiviral and anti-inflammatory effects on the influenza virus and the human respiratory syncytial virus. However, there is a lack of reports investigating the efficacy of kakkonto in RV infection. Hence, the aim of the current study was to investigate the effects of kakkonto on RV infection of human nasal epithelial (HNE) cells. HNE cells obtained via endoscopic sinus surgery were cultured and infected with RV14, with or without kakkonto treatment. The supernatants from the cells were collected, and the RV14 titer and cytokine levels were assessed. Reverse transcription-polymerase chain reaction was performed to determine the amount of viral RNA, while the level of nuclear factor kappa B (NF-κB) subunits in the nucleus was assessed by enzyme-linked immunosorbent assay. Although kakkonto treatment did not reduce RV14 titer or RNA levels, indicating that it did not inhibit RV14 proliferation, it was found to reduce the production of specific pro-inflammatory cytokines, including interleukin (IL)-8, tumor necrosis factor (TNF)-α, and monocyte chemotactic protein-1 (MCP-1). Unlike that observed with the kakkonto extract, none of the crude drugs contained in kakkonto reduced IL-8 level. Furthermore, though kakkonto treatment significantly reduced p50 levels, it did not impact the p65 subunit of NF-κB. These results indicated that kakkonto can inhibit inflammation caused by RV infection and may exert an immunomodulatory effect on HNE cells. This is the first report to elucidate the effects of kakkonto extract on RV infection in primary cultures of HNE cells, providing evidence that kakkonto may act as an effective therapy for RV infection and subsequent airway inflammation.

## Introduction

Rhinoviruses (RVs) were the most common cause of the common cold prior to the pandemic outbreak of the severe acute respiratory syndrome coronavirus 2. Specifically, RVs were reported to account for 30–50% of all respiratory illnesses in 2003 (Heikkinen and Järvinen, 2003). The common cold can cause acute exacerbation of chronic respiratory illnesses, such as bronchial asthma (BA) or chronic obstructive pulmonary disease (COPD). We have previously reported that hochuekkito, a traditional Japanese (Kampo) medicine, inhibits RV proliferation and cytokine production in human tracheal epithelial cells (Yamaya et al., 2007). Hochuekkito is commonly administered for the treatment of fatigability and anorexia. In a clinical trial, Tatsumi et al. reported that administration of hochuekkito to patients with COPD for 6 months reduced the number of common cold episodes and acute exacerbation events, improved systemic inflammation, and increased body weight (Shinozuka et al., 2007; Tatsumi et al., 2009). Other reports have indicated that hochuekkito may have a prophylactic effect on the common cold due to its immunomodulatory and immunostimulatory activities (Takayama et al., 2021). However, evidence supporting the efficacy of other Kampo medicines for the treatment of acute respiratory infections is limited.

Kakkonto (Ge-gen-tang in Chinese), one of the most frequently prescribed Kampo medicines in Japan, is used for the treatment of the common cold, shoulder stiffness, or inflammatory diseases of the upper body (DPPN, 2011). Previous studies using animal and *in vitro* models have reported the antiviral and anti-inflammatory effects of kakkonto (Arita et al., 2020). However, most experiments that have investigated the antiviral effect of kakkonto have focused on influenza viruses (Kurokawa et al., 2002; Wu et al., 2011; Shirayama et al., 2016; Geng et al., 2019) or on the human respiratory syncytial virus (Chang et al., 2012). In murine studies, administration of kakkonto has been reported to inhibit proliferation of influenza viruses in bronchoalveolar lavage fluid (Kurokawa et al., 2002) and lung tissue (Geng et al., 2019), prolong survival, and reduce mortality rates (Kurokawa et al., 2002; Geng et al., 2019). Kakkonto also reduces the expression of pro-inflammatory cytokines, including interleukin (IL)-1α, IL-6, and tumor necrosis factor (TNF)-α in H1N1 influenza-infected mice (Geng et al., 2019). Furthermore, kakkonto treatment reduces the rate of human respiratory syncytial virus infection in a human type II lung cell line (A549 cells) and human upper respiratory tract epithelial cell line (Hep-2 cells) (Chang et al., 2012). Cumulatively, these reports indicate that kakkonto might exert antiviral and anti-inflammatory effects on respiratory viral infections, although the effect of kakkonto on RV infection, both *in vivo* and *in vitro,* remains unclear. Therefore, the aim of the current study was to investigate the effect of kakkonto on RV infection of human nasal epithelial (HNE) cells, while also elucidating the underlying mechanism of action.

## Materials and Methods

### Human Nasal Epithelial Cell Culture

Nasal polyps were obtained from 22 subjects aged 55.2 ± 15.1 years with chronic rhinosinusitis who underwent endoscopic sinus surgery. Among the 22 patients, 13 (59.1%) were ex-smokers; 13 had allergic disease (nine patients with BA, three with allergic rhinitis, and one with eosinophilic sinusitis); 10 (45.5%) were prescribed nasal corticosteroids; 10 (45.5%) were treated with l-carbocisteine; 14 (63.6%) were treated with macrolides (13 patients with clarithromycin and 1 with erythromycin); 9 (40.9%) were treated with Montelukast sodium; and 9 (40.9%) were treated with antihistamine agents (Table 1). All participants provided informed consent prior to enrolment in the study. This study was approved by the Tohoku University Ethics Committee (Institutional Review Board number: 2021–1–088).

**TABLE 1:** Patient characteristics (*n* = 22).

Characteristics and medications	Value
Characteristics	
Age (years, mean ± SD)	55.2 ± 15.1
Males, *n* (%)	14 (63.6)
Smoker, *n* (%)	13 (59.1)
Brinkman index (mean ± SD)	284.8 ± 334.8
Laboratory data	
Eosinophil (%, mean ± SD)	6.8 ± 5.8
Allergic disease	
None, *n* (%)	9 (42.9)
Bronchial asthma, *n* (%)	9 (42.9)
Allergic rhinitis, *n* (%)	3 (14.3)
Eosinophilic sinusitis, *n* (%)	1 (4.5)
Medications, *n* (%)	
Inhaled corticosteroid	5 (22.7)
Nasal steroids	10 (45.5)
l-carbocisteine	10 (45.5)
Ambroxol hydrochloride	2 (9.1)
Long-acting β2 agonist	5 (22.7)
Macrolide	14 (63.6)
Montelukast sodium	9 (40.9)
Antihistamine agent	9 (40.9)

SD: standard deviation.

Isolation and culturing of HNE cells from the nasal polyps was performed as described previously (Suzuki et al., 2001; Lusamba Kalonji et al., 2015). The HNE cells were plated at a density of 7.5 × 10^5^ viable cells/mL in round-bottom plastic tubes (16 mm diameter × 125 mm length, Corning Incorporated, Corning, NY, United States) coated with human placental collagen. Cells were cultured in a 1 ml mixture of Dulbecco’s modified Eagle medium: nutrient mixture F-12 (DMEM/F-12) medium (50:50, v/v) containing 2% Ultroser G (USG) (BioSepa, Cergy-Saint-Christophe, France), 10^5^ U/L penicillin, 100 mg/L streptomycin, 5 mg/L amphotericin B, and 100 mg/L gentamicin. The tubes, loosely covered with screw caps, were placed at a 5° angle and cultured at 37°C in a 5% CO_2_ atmosphere.

### Human Embryonic Fibroblast Cell Culture

HEF cells (HEF-III cells, Riken Bio Resource Center Cell Bank, Cell No: RCB0523; Tsukuba, Japan) were cultured as previously described (Yamaya et al., 2011) with slight modifications. In brief, HEF cells were cultured in T25 flasks in Eagle’s minimum essential medium (MEM) supplemented with 10% fetal bovine serum (FBS) and antibiotics (10^5^ U/L penicillin and 100 mg/L streptomycin). The cells were then plated in 96-well plates or plastic tubes and cultured.

### Viral Stocks

A stock of clinically isolated RV14 was prepared by infecting HEF cells as described previously (Numazaki et al., 1987; Yamaya et al., 2016). Briefly, HEF cells were exposed to RV14 at 10^5^ tissue culture infective dose (TCID_50_)/mL for 1 h, rinsed with phosphate buffered saline (PBS), and cultured in plastic tubes in 1 ml MEM supplemented with 2% ultra-low IgG FBS or 10% FBS and antibiotics (10^5^ U/L penicillin and 100 mg/L streptomycin) in a 33°C incubator (HDR-6-T, Hirasawa, Tokyo, Japan) with rotation (12 rotations per hour). The cells were incubated for approximately 3 days until cytopathic effects were observed. The virus-containing fluid obtained from these cells was frozen in aliquots at −80°C.

### Preparations of Kakkonto Extract and Crude Drugs

The Japanese Pharmacopeia, 17^th^ Edition defines kakkonto as being composed of seven crude drugs: 4 g *Puerariae Radix*, 3 g *Ephedrae Herba*, 3 g *Zizyphi Fructus*, 2 g *Cinnamomi Cortex*, 2 g *Paeoniae Radix*, 2 g *Glycyrrhizae Radix*, and 2 g *Zingiberis Rhizoma* (DPPN, 2011; The Ministry of Health, Labor and Welfare, 2016). The extracts of kakkonto (lot No. 2160001010) and the seven crude drugs were obtained from Tsumura and Co., (Tokyo, Japan). The plants of origin of these crude drugs are shown in Table 2.

**TABLE 2:** Original plants of kakkonto extract used in the study.

Common name	Latin name	Scientific name of the original plants
Pueraria Root	*Puerariae Radix*	*Pueraria lobata* (Willd.) Ohwi
Ephedra Herb	*Ephedrae Herba*	*Ephedra sinica* Stapf / *Ephedra intermedia* Schrenk & C.A. Mey. / *Ephedra equisetina* Bunge
Jujube	*Zizyphi Fructus*	*Zizyphus jujuba* Mill. var. *inermis* (Bunge) Rehder
Cinnamon Bark	*Cinnamomi Cortex*	*Cinnamomum cassia* Blume
Peony Root	*Paeoniae Radix*	*Paeonia lactiflora* Pall.
*Glycyrrhiza*	*Glycyrrhizae Radix*	*Glycyrrhiza uralensis* Fisch. / *Glycyrrhiza glabra* L.
Ginger	*Zingiberis Rhizoma*	*Zingiber officinale* Roscoe

**FIGURE 1:**
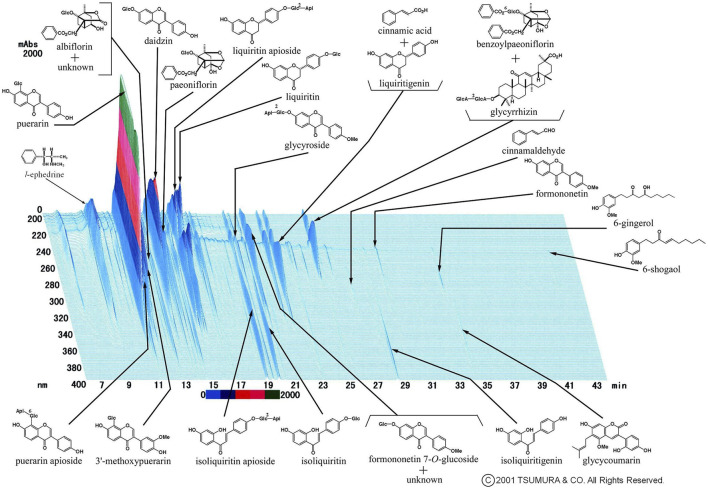
Representative 3D-HPLC image of kakkonto extract. Kakkonto contains ephedrine from *Ephedra Herba*, puerarin or daidzin from *Puerariae Radix*, paeoniflorin from *Paeoniae Radix*, cinnamic acid from *Cinnamomi Cortex*, and glycyrrhizin or liquiritin from *Glycyrrhizae Radix*.

Figure 1 shows a representative Three-Dimensional High-Performance Liquid Chromatographic(3D-HPLC) image of the kakkonto extract, provided by Tsumura and Co., (Tokyo, Japan). Due to it being composed of these seven crude drugs, Kakkonto contains many active agents, including ephedrine from the *Ephedrae Herba*, puerarin and daidzin from *Puerariae Radix*, paeoniflorin from *Paeoniae Radix*, cinnamic acid from *Cinnamomi Cortex*, and glycyrrhizin or liquiritin from *Glycyrrhizae Radix*. The CAS (Chemical Abstracts Service) registry numbers of components in kakkonto extract, shown in Figure 1, are provided as Supplementary Table.

The Japanese Pharmacopeia defines the kakkonto formula as containing the following indexed components: 9–27 mg of ephedrine or pseudoephedrine (C_10_H_15_NO: 165.23), 19–57 mg of glycyrrhizic acid (C_42_H_62_O_16_: 822.93), and 14–56 mg of paeoniflorin (C_23_H_28_O_11_: 480.46) per extract prepared according to the previously reported method (The Ministry of Health, Labor and Welfare, 2016).

The kakkonto extracts were dissolved in dimethyl sulfoxide (DMSO) at a concentration of 10 mg/ml by vortexing for 5 min at room temperature (23°C). The mixture was then dispensed in microcentrifuge tubes and frozen at −80°C until experimentation. Kakkonto stock solution was diluted in culture medium (DMEM/F-12 with 2% USG) at a concentration of 100 μg/ml and the concentrations of ephedrine, pseudoephedrine, and glycyrrhizic acid were determined using liquid chromatography-tandem mass spectrometry (LC-MS/MS) performed by Sekisui Chemistry, Tokyo, Japan.

### Rhinovirus Infection and Treatment

Infection of HNE cells with RV14 was performed using previously described methods (Yamaya et al., 2007). A stock solution of RV was added to the cells in round-bottom plastic tubes (1 ml in each tube, 1.0 × 10^5^ TCID_50_/mL, 0.13 TCID_50_/cell of the multiplicity of infection). After 1 h of incubation, the viral solution was removed, and cells were rinsed with PBS. The cells were then cultured in 1 ml fresh medium (2% USG in DMEM/F12) in the presence, or absence, of kakkonto extracts diluted in culture medium. The kakkonto treatments were initiated 1 h after infection and continued until the end of the experimental period. To investigate the concentration-dependent effects, cells were treated with 10, 20, or 50 μg/ml kakkonto. The supernatants (1 ml) were collected before infection, as well as 1, 3, 5, and 7 days after infection, and rapidly frozen in ethanol at −80°C. The specific kakkonto concentrations used in this study were determined based on the detected concentrations of ephedrine/pseudoephedrine and glycyrrhizic acid in the kakkonto solution. The specific details of these calculations can be found in *Concentration of Index Components in Kakkonto Extracts*.

### Detection and Titration of Viruses

The detection and titration of RV14 in culture supernatants were performed using the endpoint method by infecting proliferating HEF cells in plastic 96-well plates containing 10-fold dilutions of virus-containing supernatants with MEM supplemented with 2% ultra-low IgG calf serum and antibiotics (10^5^ U/L penicillin and 100 mg/L streptomycin), as described previously (Suzuki et al., 2001; Yamaya et al., 2014). The typical cytopathic effects of RV were then examined. The viral titers in the supernatants were expressed as TCID_50_/mL.

### Quantification of Rhinovirus RNA

The levels of RV14 RNA were determined using quantitative reverse transcription-polymerase chain reaction (RT-PCR) with TaqMan gene expression master mix (Applied Biosystems, Foster City, CA, United States), as described previously (Yamaya et al., 2011). The amount of RV14 RNA was normalized to that of β-actin, which was used as a housekeeping gene.

### Determination of Cytokine Concentration

IL-6 and IL-8 level were assessed using an enzyme-linked immunosorbent assay (ELISA) performed by LSI Medience Corporation (Tokyo, Japan) with a solid phase chemiluminescent ELISA kit (QuantinGlo®; ELISA, R&D Systems, MN, United States), and an IL-8 EIA kit (Invitrogen, CA, United States). The levels of other cytokines were determined by a multiplex suspension array (Genetic Lab Co., Ltd., Sapporo, Japan) using a Luminex® assay system (Luminex Corp., Austin, TX, United States) with a magnetic Luminex® assay human premixed multi-analyte kit (R&D Systems Inc., Minneapolis, MN, United States). The assay was performed according to the manufacturer’s instructions. Briefly, all samples were diluted using Calibrator Diluent RD6-52, and 50 μL of the diluted samples were used for the assay customized to detect and quantify TNF-α, IL-1β, IL-17C, IL-17E/IL25, granulocyte-macrophage colony-stimulating factor (GM-CSF), monocyte chemotactic protein-1 (MCP-1), thymic stromal lymphopoietin (TSLP), thymus and activation-regulated chemokine (TARC), eotaxin, and regulated on activation normal T cell expressed and secreted (RANTES). The fluorescence intensity of each sample was measured using a Luminex® 100/200TM system (Luminex Corp., Austin, TX, US), and the concentrations of each analyte were calculated using MILLIPLEX® Analyst 5.1 (Merck Millipore Corp., Burlington, MA, United States).

### Measurement of Changes in Acidic Endosomes

The distribution and fluorescence intensity of acidic endosomes in the cells were measured with LysoSensor DND-189 dye (Molecular Probes, Eugene, OR, United States) using live-cell imaging (Lusamba Kalonji et al., 2015). The cells on coverslips in Petri dishes were observed under a fluorescence microscope (Olympus IX70; Olympus Co., Ltd., Tokyo, Japan). The excitation wavelength was 443 nm, and the light emitted from the cells was detected using a 505 nm filter. Fluorescence intensity was calculated using a fluorescence image analyzer system (Lumina Vision®; Mitani Co. Ltd., Fukui, Japan) equipped with a fluorescence microscope. HNE cells were treated with kakkonto (20 μg/ml) or media alone. The fluorescence intensity of the acidic endosomes was measured in 100 cells, and the mean value of the fluorescence intensity was expressed as a percentage of the control value compared to the fluorescence intensity of the cells prior to treatment.

### Assessment of NF-κB Activation

Nuclear proteins were extracted from the nuclear extract kit of the epithelial cells (Active Motif, Carlsbad, CA, United States). The presence of translocated p50 and p65 subunits in the nuclear extracts was assessed using a TransAM NFκB family kit (Active motif, Carlsbad, CA, United States), according to the manufacturer’s instructions. To examine the effect of kakkonto on the NF-κB subunits in the HNE cells, the cells were pretreated with kakkonto (20 μg/ml) or medium alone at 33°C for 24 h, after which the nuclear proteins were extracted.

### Statistical Analysis

Results were expressed as mean ± standard error (SE). Statistical analysis was performed using two-way repeated-measures analysis of variance (ANOVA). Student’s *t*-test or Mann-Whitney U-test was used for comparison between two groups according to the distribution patterns of the evaluated variables. Statistical significance was set at *p* < 0.05. In all experiments, n refers to the number of donors from whom the epithelial cells were obtained. All analyses were performed using SPSS version 21 (IBM Japan, Tokyo, Japan).

## Results

### Concentration of Index Components in Kakkonto Extracts

LC-MS/MS analysis revealed that the concentrations of ephedrine/pseudoephedrine and glycyrrhizic acid in the kakkonto solution (100 μg/ml) were 363.0 ± 13.9 ng/ml and 867.3 ± 22.2 ng/ml, respectively. We then estimated the concentration of ephedrine/pseudoephedrine in human serum after administration of kakkonto to be up to 40–60 ng/ml (Yafune and Cyong, 2001; Inotsume et al., 2009), and that of glycyrrhetinic acid to be 100 ng/ml (provided by Tsumura and Co.,) based on a previous study (Yamaya et al., 2007). The 40–60 ng/ml of ephedrine/pseudoephedrine was calculated to apply 11.0–16.5 μg/ml of kakkonto extract and 100 ng/ml of glycyrrhetinic acid was 20.2 μg/ml of kakkonto extract. Therefore, we determined the concentration of kakkonto extract to be 10 or 20 μg/ml in our experiments.

### Effects of Kakkonto on Rhinoviral Replication

**FIGURE 2:**
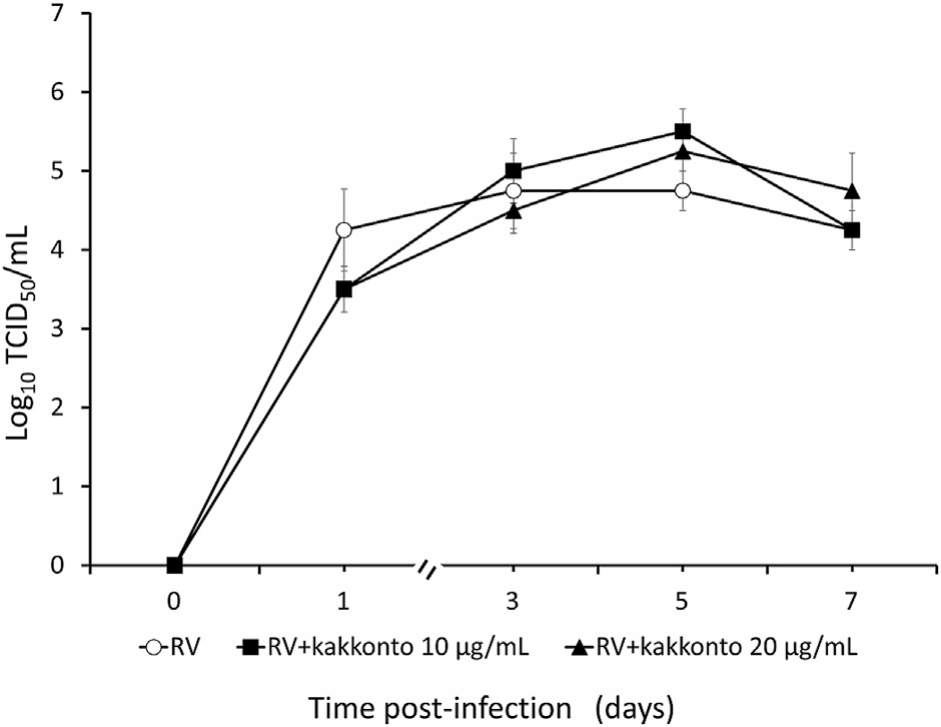
Time course of viral release into supernatants of HNE cells infected with RV14 and treated with kakkonto (10 μg/ml, closed squares), kakkonto (20 μg/ml, closed triangles), or medium only (open circles). Results are presented as the mean ± standard error from four subjects.

**FIGURE 3:**
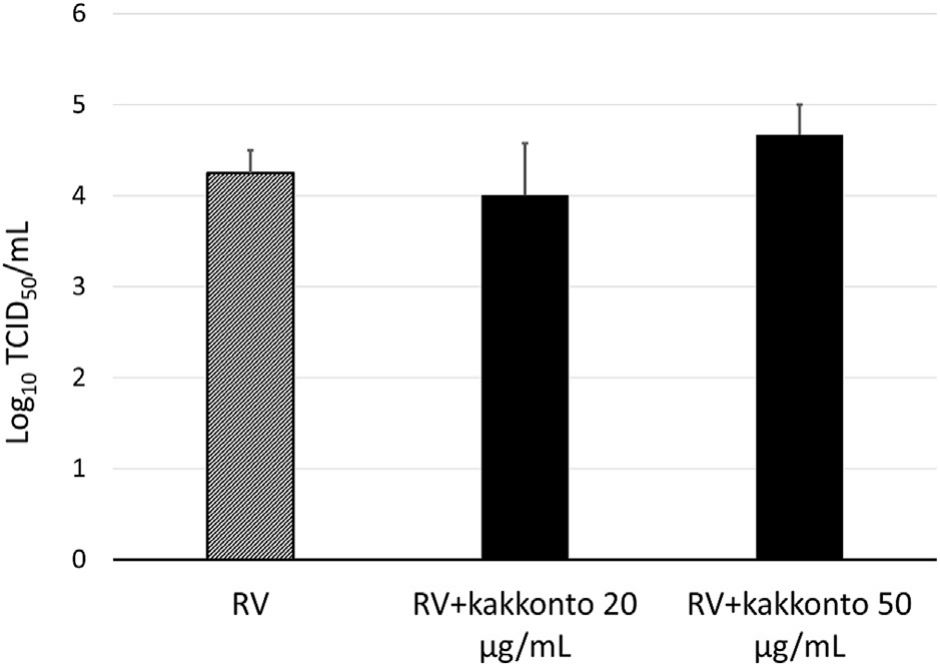
Viral release into supernatants of HNE cells treated with 20 μg/ml and 50 μg/ml kakkonto or cultured in medium alone and collected 24 h after RV infection. Results are presented as the mean ± standard error from four subjects.

The RV14 titer increased significantly until 7 days after infection (*p* = 0.001; Figure 2). Treatment of HNE cells with 10 and 20 μg/ml kakkonto 1 h after infection did not reduce RV titers (*p* = 0.36). However, the RV titer in the supernatants of the cells treated with kakkonto tended to decrease slightly by 24 h post-infection (h.p.i.). We then measured viral titers in the supernatants of HNE cells treated with 50 μg/ml kakkonto (Figure 3) and found that RV14 titers in the supernatants collected 24 h.p.i. did not differ significantly from that of cells cultured in medium alone or treated with 20 μg/ml kakkonto. These results indicate that kakkonto extract did not inhibit RV proliferation.

### Effects of Kakkonto on RV14 RNA Expression

**FIGURE 4:**
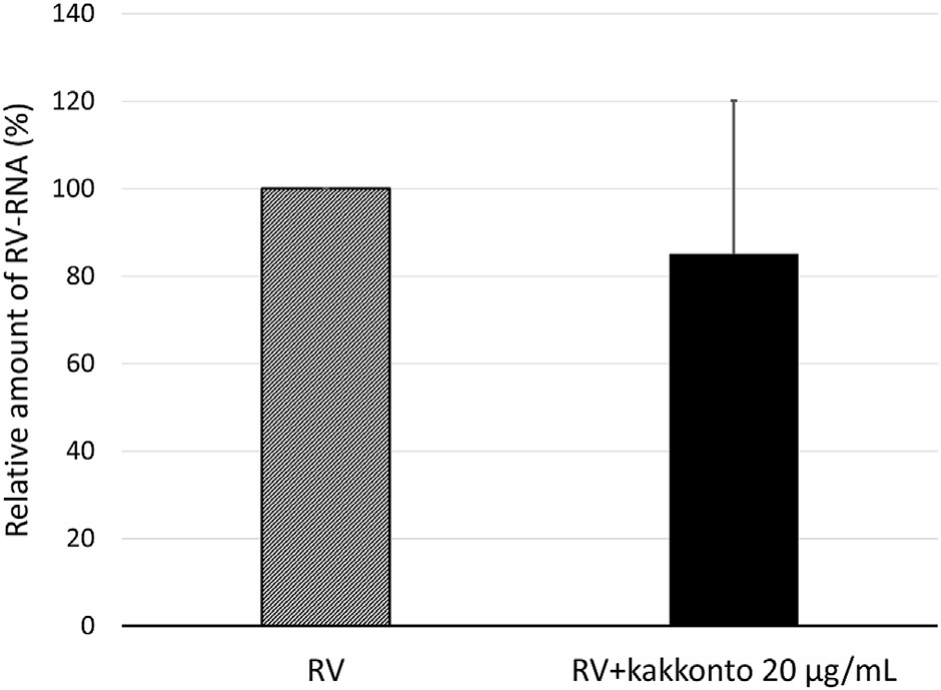
Relative amount of RV14 RNA in HNE cells treated with 20 μg/ml kakkonto compared to cells cultured in medium alone 24 h after infection. Results are expressed as the relative amount (%) compared to that of cells cultured in medium alone and presented as mean ± standard error from five subjects. Kakkonto treatment did not reduce the relative amount of RV14 RNA (*p* = 0.69).

To investigate the effect of kakkonto on the proliferation of RV14, we determined the level of RV14 RNA in the cells 1 day post-infection (d.p.i., Figure 4). Treatment with 20 μg/ml kakkonto tended to decrease the amount of RV14 RNA; however, the difference between kakkonto treatment and medium alone was not statistically significant (*p* = 0.69).

### Effects of Kakkonto on Endosome Acidification

**FIGURE 5:**
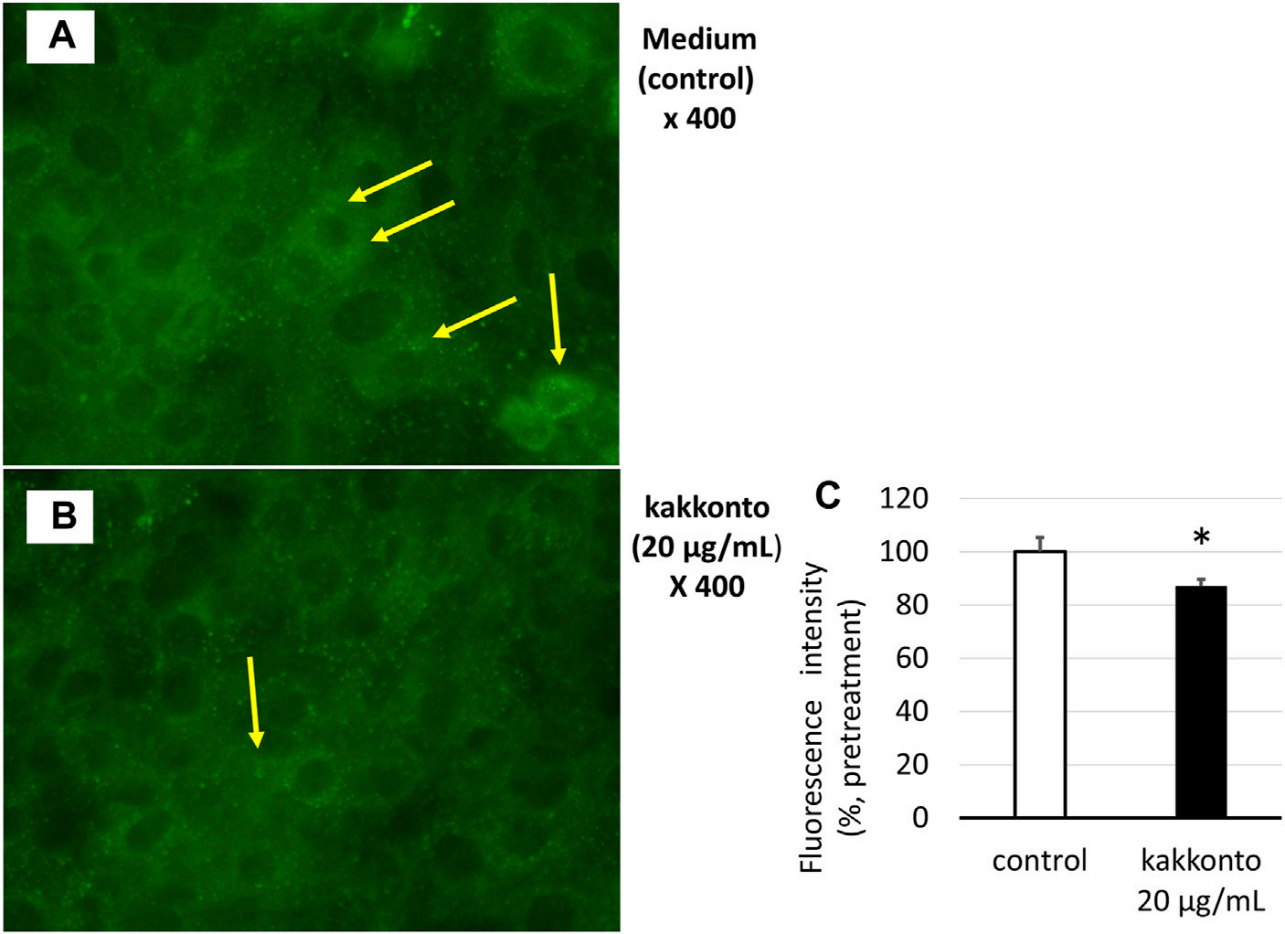
**(A,B)** Distribution of acidic endosomes exhibiting green fluorescence in HNE cells 24 h after treatment with kakkonto (20 μg/ml) **(B)** or medium **(A)**. Yellow arrows indicate acidic endosomes in HNE cells. Data are representative of four individual experiments. Magnification, ×400. **(C)** Fluorescence intensity of acidic endosomes in HNE cells 24 h after treatment with kakkonto (20 μg/ml) or medium (control). The mean fluorescence intensity of the control cells was set at 100%. *p < 0.05.

The treatment of HNE cells with 20 μg/ml kakkonto for 24 h significantly reduced the number of green-fluorescent acidic endosomes (Figures 5A,B), as well as the fluorescence intensity of acidic endosomes compared to that of cells cultured in medium alone (*p* = 0.027, Figure 5C).

### Effects of Kakkonto on Cytokine Production

**FIGURE 6:**
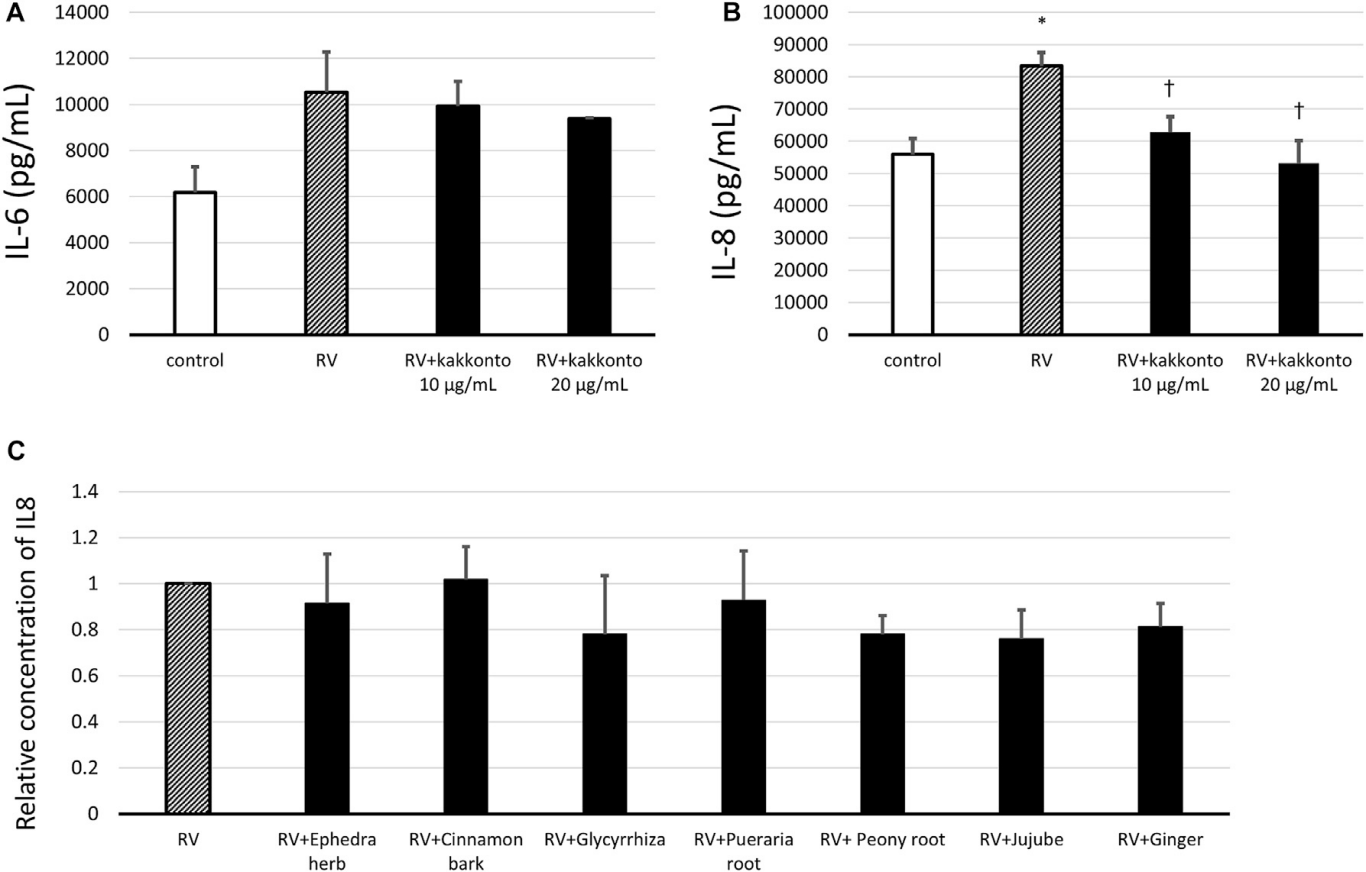
**(A,B)** Release of IL-6 **(A)** and IL-8 **(B)** into culture supernatants 24 h after RV14 infection in the presence of 10 μg/ml or 20 μg/ml kakkonto, or after culturing in medium alone (RV) or mock infection (control). *p < 0.05 significant differences compared to control cells in the absence of RV infection (control); **(B)**. †*p* < 0.05 significant differences compared to RV infection alone (RV). **(C)** Relative abundance of IL-8 released in culture supernatants 24 h after RV14 infection in the presence of each crude drug of the kakkonto extract. Results are expressed as the relative amount (%) compared to cells cultured in medium alone (RV) and are presented as the mean ± SE from three **(A,B)** and seven **(C)** HNE cultures. The concentration of each crude drug was calculated as the weight % of 20 μg/ml kakkonto extract.

The concentration of IL-6 in the supernatants of untreated, infected cells increased 24 h.p.i. (Figure 6A); whereas treatment with kakkonto tended to reduce IL-6 levels in a dose-dependent manner 24 h.p.i., however, this reduction was not statistically significant. We also measured the concentration of IL-6 3 d.p.i and found the concentration of IL-6 in the supernatants of uninfected cells (control), untreated infected cells, and kakkonto-treated infected cells to be 9,027 ± 1,260, 9,773 ± 1,026, and 7,933 ± 570 pg/ml, respectively. Kakkonto treatment tended to reduce the level of IL-6 compared to that in the supernatants of the cells cultured in medium alone, although this reduction was not statistically significant (*p* = 0.19).

In untreated, RV-infected cells, we also observed that the level of IL-8 significantly increased 24 h.p.i. compared to uninfected controls (*p* = 0.013; Figure 6B). Meanwhile, treatment with 10 or 20 μg/ml kakkonto significantly reduced IL-8 production compared to the untreated, infected cells (*p* = 0.031 and 0.021, respectively). We also assessed IL-8 concentration 3 d.p.i. and found it to 142,667 ± 1,453, 149,333 ± 7,216, and 122,000 ± 7,638 pg/ml in the supernatants collected from uninfected cells, infected and untreated cells, and infected cells treated with kakkonto (20 μg/ml), respectively. Thus, 20 μg/ml kakkonto treatment tended to reduce IL-8 concentration 3.d.p.i compared to RV14 infection alone, although the difference was not statistically significant (*p* = 0.059).

We also assessed the IL-8 level in the supernatant of cells 24 h.p.i. treated with individual extracts of the crude drugs (Figure 6C). The concentration of each crude drug was calculated as weight percentage of 20 μg/ml of kakkonto extract; that is 4 μg/ml of *Puerariae Radix*, 3 μg/ml of *Ephedrae Herba* and *Zizyphi Fructus*, and 2 μg/ml of *Cinnamomi Cortex*, *Glycyrrhizae Radix*, *Paeoniae Radix*, and *Zingiberis Rhizoma*, were applied. However, none of the crude drugs significantly reduced IL-8 concentration in the supernatants of RV14-infected cells (Figure 6C).

Furthermore, RV14 infection was found to increase the concentration of TNF-α, IL-1β, GM-CSF, and MCP-1 (Table 3), while kakkonto treatment (20 μg/ml) tended to reduce these effects. However, these differences were not statistically significant due to the relatively large standard errors (SE). We, therefore, elected to re-analyze this data by comparing concentrations to those of uninfected control supernatants (Table 4). RV14 infection was found to not affect the supernatant concentration of IL-17C, IL-25, or TARC. Meanwhile, TNF-α and MCP-1 levels in RV14-infected samples treated with kakkonto were significantly lower than those in untreated, RV-infected cultures (*p* = 0.036 and 0.033, respectively). RV infection also increased the levels of IL1β, GM-CSF, TSLP, while kakkonto treatment only slightly reduced these effects, however, the differences were not statistically significant. Also, RV infection tended to increase eotaxin levels, however, kakkonto treatment did not impact these effects. The levels of RANTES were below the level of detection in seven of the nine samples.

**TABLE 3:** Cytokine concentration in the supernatants of HNE cells 24 h after RV infection.

Cytokines and chemokines	Control (*n* = 3)	RV (*n* = 3)	RV + kakkonto (*n* = 3)
TNF-α (pg/mL, mean ± SE)	8.2 ± 2.9	9.8 ± 4.4	5.2 ± 1.2
* p* value vs. control	―	0.79	0.39
* p* value vs. RV	―	―	0.41
IL-1β (pg/mL, mean ± SE)	8.1 ± 3.8	19.6 ± 15.5	12.4 ± 8.6
* p* value vs. control	―	0.83	0.83
* p* value vs. RV	―	―	0.83
GM-CSF (pg/mL, mean ± SE)	13.7 ± 3.4	19.2 ± 7.7	9.8 ± 2.5
* p* value vs. control	―	0.55	0.40
* p* value vs. RV	―	―	0.31
MCP-1 (pg/mL, mean ± SE)	1,170 ± 349	1,372 ± 537	628 ± 167
* p* value vs. control	―	0.77	0.24
* p* value vs. RV	―	―	0.26
IL-17C (pg/mL, mean ± SE)	122.0 ± 31.3	125.0 ± 51.9	124.2 ± 70.9
* p* value vs. control	―	0.56	0.70
* p* value vs. RV	―	―	0.31
IL-25 (pg/mL, mean ± SE)	89.0 ± 0.0	90.5 ± 8.0	90.4 ± 3.5
* p* value vs. control	―	0.86	0.74
* p* value vs. RV	―	―	0.99
TSLP (pg/mL, mean ± SE)	1.5 ± 0.2	1.7 ± 0.2	1.4 ± 0.1
* p* value vs. control	―	0.51	0.83
* p* value vs. RV	―	―	0.27
TARC (pg/mL, mean ± SE)	54.5 ± 1.8	56.5 ± 3.7	57.5 ± 3.0
* p* value vs. control	―	0.82	0.49
* p* value vs. RV	―	―	0.64
Eotaxin (pg/mL, mean ± SE)	22.3 ± 2.1	25.4 ± 4.6	24.9 ± 2.3
* p* value vs. control	―	0.64	0.49
* p* value vs. RV	―	―	1.0

RANTES data is not presented as it was below the level of detection in seven of nine samples.

HNE, human nasal epithelial; RV, Rhinovirus; SE, standard error; TNF, tumor necrosis factor; IL, interleukin; GM-CSF, granulocyte-macrophage colony-stimulating factor; MCP-1, monocyte chemotactic protein-1; TSLP, thymic stromal lymphopoietin; TARC, thymus and activation-regulated chemokine; RANTES, regulated on activation normal T cell expressed and secreted.

**TABLE 4:** Cytokine concentration in the supernatants of HNE cells compared to control samples 24 h after RV infection.

Cytokines and chemokines	Control (*n* = 3)	RV (*n* = 3)	RV + kakkonto (*n* = 3)
TNF-α (means ± SE)	1	1.11 ± 0.12	0.69 ± 0.07
* p* value vs. control	–	0.38	0.051
* p* value vs. RV	–	–	0.036
IL-1β (means ± SE)	1	1.73 ± 0.77	1.22 ± 0.34
* p* value vs. control	–	0.44	0.57
* p* value vs. RV	–	–	0.58
GM-CSF (means ± SE)	1	1.31 ± 0.23	0.72 ± 0.05
* p* value vs. control	–	0.30	0.028
* p* value vs. RV	–	–	0.12
MCP-1 (means ± SE)	1	1.14 ± 0.18	0.56 ± 0.04
* p* value vs. control	–	0.47	0.009
* p* value vs. RV	–	–	0.033
IL-17C (means ± SE)	1	0.96 ± 0.20	0.93 ± 0.35
* p* value vs. control	–	0.49	0.49
* p* value vs. RV	–	–	0.51
IL-25 (means ± SE)	1	1.02 ± 0.09	1.01 ± 0.04
* p* value vs. control	–	0.86	0.74
* p* value vs. RV	–	–	0.99
TSLP (means ± SE)	1	1.13 ± 0.02	0.95 ± 0.07
* p* value vs. control	–	0.027	0.52
* p* value vs. RV	–	–	0.067
TARC (means ± SE)	1	1.04 ± 0.07	1.06 ± 0.06
* p* value vs. control	–	0.62	0.41
* p* value vs. RV	–	–	0.84
Eotaxin (means ± SE)	1	1.16 ± 0.21	1.16 ± 0.23
* p* value vs. control	–	0.53	0.56
* p* value vs. RV	–	–	1.00

Data are expressed as the ratio of cytokine concentration in the supernatants of RV-infected HNE cells to that in uninfected HNE cells (control).

HNE, human nasal epithelial; RV, Rhinovirus; SE, standard error; TNF, tumor necrosis factor; IL, interleukin; GM-CSF, granulocyte-macrophage colony-stimulating factor; MCP-1, monocyte chemotactic protein-1; TSLP, thymic stromal lymphopoietin; TARC, thymus and activation-regulated chemokine; RANTES, regulated on activation normal T cell expressed and secreted.

### Effects of Kakkonto on NF-κB

**FIGURE 7:**
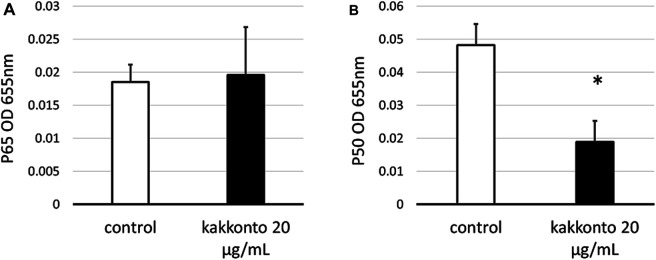
Amount of p65 **(A)** and p50 **(B)** in the nuclear extracts of cells treated with or without 20 μg/ml kakkonto extract. ^*^p < 0.05 significant difference compared to control cells. Results are expressed as optical density and presented as mean ± SE from five HNE cultures.

To investigate the mechanism underlying kakkonto-induced cytokine production, we quantified the nuclear levels of NF-κB subunits (p65 and p50) in HNE cells. Compared to the untreated control, Kakkonto treatment (20 μg/ml) did not impact the levels of p65 (Figure 7A) but significantly reduced the levels of p50 (Figure 7B) 24 h.p.i. (*p* < 0.05).

## Discussion

In the present study, we investigated the effect of kakkonto extract on RV infection in primary cultures of HNE cells. Kakkonto treatment did not reduce the RV14 titers or RNA levels, however, it did significantly reduce the number, and fluorescence intensity of, acidic endosomes, through which RV RNA enters the cytoplasm of epithelial cells (Perez and Carrasco 1993; Casasnovas and Springer 1994). These results indicate that the effect of kakkonto on the modulation of endosomal pH might not be sufficient for inhibiting RV14 replication.

RV infection induces the production of various cytokines, including IL-1β, IL-6, IL-8, TNF-α, GM-CSF, and MCP-1 (Message and Johnston, 2004; Jacobs et al., 2013; Schuler et al., 2014). These pro-inflammatory cytokines and chemokines play important roles in the subsequent innate and adaptive immune responses. Despite their beneficial effects on viral clearance from the respiratory tract, the generation of pro-inflammatory mediators and the recruitment of inflammatory cells results in immunopathological changes in the in airway and may exacerbate airway inflammation (Message and Johnston, 2004). In the current study we observed that kakkonto treatment decreased production of certain cytokines, namely, IL-8, TNFα, and MCP-1 at 24 h. p.i., whereas it did not significantly reduce the enhanced IL-6 level post-infection. Hence, Kakkonto extract may exert an inhibitory effect on airway inflammation induced by RV infection.

IL-8 plays an important role in neutrophil migration to sites of inflammation. In fact, IL-8 concentration in sputum is reportedly related to the severity of COPD and BA, as well as accelerated reduction in forced expiratory volume (Shannon et al., 2008; Dong et al., 2020; Marc-Malovrh et al., 2020). Meanwhile, IL-8 is not only associated with neutrophilic, but also eosinophilic inflammation, as IL-8-stimulated neutrophils lead to accumulation of eosinophils (Kikuchi et al., 2006; Nakagome and Nagata, 2018). IL-8 level was markedly reduced when HNE cells were treated with kakkonto extract. Thus, considering that the concentration of extracellular IL-8 was markedly reduced following treatment of RV-infected HNE cells with kakkonto extract, kakkonto may alleviate both neutrophilic and eosinophilic inflammation in RV-infected airways.

RV14, the virus employed in the current study, represents a major-group RV. This virus uses intercellular adhesion molecule-1 as a cell surface receptor for entry. After adhesion, viral antigen recognition occurs via pattern recognition receptors in airway epithelial cells. Toll-like receptor (TLR) 2, on the cell surface, recognizes viral capsid proteins. After entering endosomes, TLR 3 and TLR 7/8 bind to double-and single-stranded RNA, respectively. Interactions with these TLRs trigger activation of the NF-κB pathway and increase production of interferon (IFN)-β, IFN-γ, IL-6, and IL-8 (Jacobs et al., 2013; Makris and Johnston, 2018; Ganjian et al., 2020). Geng et al. reported that kakkonto treatment decreases the mRNA and protein levels of TLR7 and myeloid differentiation primary response 88 in murine lung tissues infected with H1N1 influenza virus, indicating that kakkonto exerts an inhibitory effect on activation of the NF-κB pathway (Geng et al., 2019). Hence, kakkonto treatment, in the current study, was predicted to decrease the abundance of NF-κB subunits, considering the reduction in IL-8 and TNF-α levels in the supernatants of HNE cells. However, although significant reduction was observed in the number of p50 subunits, no effect was observed on the expression of p65. Additionally, levels of IL-6, MCP-1, and IL-1β were slightly reduced following treatment of RV-infected cells with kakkonto. However, considering that the patients providing HNE cells in this study were treated with inhaled corticosteroids (22.7%), nasal steroids (45.5%), l-carbocisteine (45.5%), ambroxol hydrochloride (9.1%), long-acting β2 agonists (22.7%), or macrolides (63.6%), the use of these drugs may have influenced the RV titer or cytokine levels, as well as the abundance of NF-κB. Indeed, these drugs were previously reported to exert antiviral and anti-inflammatory effects against RV infection (Suzuki et al., 2002; Yamaya et al., 2011; Yamaya et al., 2014; Yamaya et al., 2016). In particular, the high rate of treatment using nasal steroids may affect the levels of cytokines associated with eosinophilic inflammation, such as IL-25, TALC, and eotaxin, which were not affected by RV infection or kakkonto treatment. In contrast, the results of the present study reflected the clinical response of patients with chronic sinusitis or BA infected with RV. Hence, kakkonto may be useful in inhibiting further inflammation when these patients contract common cold.

Kakkonto is composed of seven crude drugs obtained from *Puerariae Radix, Ephedrae Herba, Zizyphi Fructus*, *Cinnamomi Cortex, Paeoniae Radix, Glycyrrhizae Radix*, and *Zingiberis Rhizoma*. The antiviral and anti-inflammatory effects of each crude drug have been investigated to some extent. For example, Chang et al. reported that ginger (*Zingiber officinale*) and *Paeonia lactiflora* inhibit human respiratory syncytial virus-induced plaque formation in the airway epithelium by blocking viral attachment and internalization (Chang et al., 2013; Lin et al., 2013). Nomura et al. showed that influenza viral release is inhibited by *Glycyrrhizae Radix* extract (Nomura et al., 2019). Meanwhile, *Cinnamomum* possesses antiviral, antibacterial, antioxidant, and anti-inflammatory effects (Kumar et al., 2019). Similarly, glycyrrhizin, the major component of *Glycyrrhizae Radix*, has been reported to exert anti-inflammatory and antiviral effects on respiratory viruses, hepatitis viruses, and human immunodeficiency virus (Fiore et al., 2008; Sun et al., 2019). Moreover, puerarin, one of the components of *Pueraria Radix*, may have an inhibitory effect on oxidative stress and apoptosis (Wei et al., 2014). We previously reported that glycyrrhizin reduces RV release in the supernatants of human tracheal epithelial cells (Yamaya et al., 2007). Meanwhile, in the current study, although kakkonto extract decreased the concentration of IL-8 in the supernatants of RV-infected HNE cells, none of the crude drugs present in kakkonto significantly impacted IL-8 levels. These results suggest that the inhibitory effects of kakkonto extracts on IL-8 production may be due to the overall effect of its constituent crude drugs. However, the antiviral and anti-inflammatory effects of these crude drugs, as well as their chemical components, and synergistic action, have not been sufficiently elucidated and require further investigation to confirm their efficacy.

Certain limitations were noted in this study. First, we did not investigate the effects of each chemical component of kakkonto (e.g., ephedrine, puerarin, glycyrrhizin, cinnamaldehyde, and paeoniflorin). Meanwhile, Wang et al. reported that puerarin inhibits the expression of TNF-α, IL-6, and IL-1β in LPS-induced murine lung tissue and reduces IL-8 production in A549 cells (Wang et al., 2018). Glycyrrhizin also inhibits IL-8 production and NF-κB activity in lung epithelial cells (Takei et al., 2008). Considering none of the crude drugs significantly decreased the IL-8 levels after RV infection, we speculated that these chemical components may also exert cumulative effects on inflammation or immunoreaction after RV infection. Second, we used 10–20 μg/ml kakkonto, as serum ephedrine and pseudoephedrine concentrations may be 40–60 ng/ml after administration of the kakkonto extract; whereas most previous *in vitro* studies used > 30–300 μg/ml kakkonto (Chang et al., 2012; Kitamura et al., 2014; Geng et al., 2019). Hence, more significant results may have been attained had we employed higher concentrations of kakkonto. Nevertheless, along with the primary culture of HNE cells, the concentrations adopted in this study are likely more reflective of the true physiological conditions of the human body. Finally, the mechanisms of the anti-inflammatory effect of kakkonto were not sufficiently investigated. A previous study indicated that production of IL-8 is also associated with NF-κB p50 activation (Das et al., 2012). Kakkonto might contribute to the inhibition of cytokine production by reducing NF-κB activation, although why it did not inhibit NF-κB p65 activation is unclear. There is a need to evaluate the expression levels of proteins relevant to the signaling pathway of NF-κB, such as p-p65, inhibitor-of-kappa-B proteins (IκB), or IκB kinase. However, the measurements of these proteins have been performed by using murine lung tissue or human cell lines thus far (Zhou et al., 2017; Geng et al., 2019; Ma et al., 2021). Further examination using primary cultures of HNE cells is needed to analyze these factors.

The severity of viral infection reflects the regulation of virus proliferation and the extent of virus-associated inflammation (Lauder et al., 2013). Until now, more than 160 RV serotypes have been identified (Basnet et al., 2019). Owing to little cross-neutralization among serotypes, no RV vaccine has yet been established, and no antiviral therapeutics for treating RV infections are available (Jacobs et al., 2013). Here, we elucidated that kakkonto may exert anti-inflammatory and immune-modulatory effects on RV-infected cells, indicating that kakkonto may be used as a treatment strategy for RV infection.

The present study revealed that kakkonto may exert an anti-inflammatory effect following RV infection via suppression of pro-inflammatory cytokine production. Specifically, kakkonto may reduce inflammation induced by RV infection, thereby reducing chronic airway inflammation. To our knowledge, this is the first study to elucidate the effects of kakkonto extract on RV infection in primary cultures of HNE cells. However, further investigation is needed to elucidate the detailed mechanism of the anti-inflammatory effects and clinical efficacy of kakkonto in chronic respiratory diseases.

## Data Availability

The raw data supporting the conclusions of this article will be made available by the authors, without undue reservation.

## Abbreviations

BA, bronchial asthma; COPD, chronic obstructive pulmonary disease; GM-CSF, Granulocyte-macrophage colony-stimulating factor; HNE, human nasal epithelial cells, HEF, human embryonic fibroblasts; IL, interleukin; MCP-1, monocyte chemotactic protein-1; RT-PCR, reverse transcription-polymerase chain reaction; RV, Rhinovirus; TNF, tumor necrosis factor.
