# Supplementary material for: Kakkonto Inhibits Cytokine Production Induced by Rhinovirus Infection in Primary Cultures of Human Nasal Epithelial Cells
Source: Front Pharmacol. 2021 Aug 31;12:687818. doi: 10.3389/fphar.2021.687818 (PMC8438568; doi:10.3389/fphar.2021.687818)
Supplement: Supplementary file 1 [file Table1.docx]

Supplementary Material

# SupplementaryTable

CAS registry numbers of the chemical components of kakkonto extract

| Name of chemical substance | CAS registry number |
| --- | --- |
| 3'-methoxypuerarin | 117047-07-1 |
| 6-gingerol | 39886-76-5 / 58253-27-3 |
| 6-shogaol | 555-66-8 / 23513-13-5 |
| albiflorin | 39011-90-0 |
| benzoylpeoniflorin | 38642-49-8 |
| cinnamaldehyde | 104-55-2 / 14371-10-9 |
| cinnamic acid | 140-10-3 / 621-82-9 |
| daidzin | 552-66-9 |
| formononetin 7-O-glucoside | 486-62-4 |
| formononetin | 485-72-3 |
| glycycoumarin | 94805-82-0 |
| glycyroside | 125310-04-5 |
| glycyrrhizin | 1405-86-3 |
| isoliquiritigenin | 961-29-5 / 13745-20-5 |
| isoliquiritin | 5041-81-6 / 7014-39-3 |
| isoliquiritin apioside | 120926-46-7 |
| l-ephedrine | 299-42-3 |
| liquiritigenin | 578-86-9 |
| liquiritin apioside | 74639-14-8 |
| liquiritin | 551-15-5 |
| peoniflorin | 23180-57-6 |
| puerarin apioside | 103654-50-8 |
| puerarin | 3681-99-0 |

CAS: Chemical Abstracts Service
